# Distinct and overlapping functions of glutathione peroxidases 1 and 2 in limiting NF-κB-driven inflammation through redox-active mechanisms

**DOI:** 10.1016/j.redox.2019.101388

**Published:** 2019-11-16

**Authors:** Solveigh C. Koeberle, André Gollowitzer, Jamila Laoukili, Onno Kranenburg, Oliver Werz, Andreas Koeberle, Anna P. Kipp

**Affiliations:** a Department of Molecular Nutritional Physiology, Institute of Nutritional Sciences, Friedrich Schiller University Jena, Germany; b Department of Pharmaceutical/Medicinal Chemistry, Institute of Pharmacy, Friedrich Schiller University Jena, Germany; c Department of Surgery, University Medical Center Utrecht, Utrecht, the Netherlands; d Michael Popp Research Institute, University of Innsbruck, Austria; e TraceAge-DFG Research Unit on Interactions of Essential Trace Elements in Healthy and Diseased Elderly, Potsdam-Berlin-Jena, Germany

**Keywords:** Glutathione peroxidase, Inflammation, NF-κB, Prostaglandins, Lipid mediators, Inflammatory bowel disease, AA, arachidonic acid, COX, cyclooxygenase, DHA, docosapentaenoic acid, DHR123, dihydrorhodamin, DSS, dextran sodium sulfate, EPA, eicosapentaenoic acid, GPx, glutathione peroxidase, HDHA, hydroxy-docosahexaenoic acid, HEPE, hydroxy-eicosapentaenoic acid, HETE, hydroxy-eicosatetraenoic acid, HHT, hydroxy-heptadecatrienoic acid, HODE, hydroxy-octadecadienoic acid, HPETE, hydroxy-eicosatetraenoic acid, HPODE, hydroxy-octadecadienoic acid, IKK, IκB kinase, IL, interleukin, iNOS, inducible NO synthase, IκBα, inhibitor of NF-κB, kd, knockdown, KO, knockout, LM, lipid mediators, LOX, lipoxygenase, LT, leukotriene, mPGES1, microsomal prostaglandin E_2_ synthase 1, NAC, *N*-acetyl cysteine, NF-κB, nuclear factor 'kappa-light-chain-enhancer’ of activated B-cells, NOX, NADPH oxidase, PG, prostaglandin, ROS, reactive oxygen species, scr, scramble, TBHP, tert-butyl hydroperoxide, TNF, tumor necrosis factor, WT, wild type

## Abstract

Glutathione peroxidase 2 (GPx2) is one of the five selenoprotein GPxs having a selenocysteine in the active center. GPx2 is strongly expressed in the gastrointestinal epithelium, as is another isoform, GPx1, though with a different localization pattern. Both GPxs are redox-active enzymes that are important for the reduction of hydroperoxides.

Studies on GPx2-deficient mice and human HT-29 cells with a stable knockdown (kd) of GPx2 revealed higher basal and IL-1β-induced expression of NF-κB target genes *in vivo* and *in vitro*. The activation of the IKK–IκBα–NF-κB pathway was increased in cultured GPx2 kd cells. Basal signaling was only restored by re-expressing active GPx2 in GPx2 kd cells but not by redox-inactive GPx2. As it is still not clear if the two isoforms GPx1 and GPx2 have different functions, kd cell lines for either GPx1 or GPx2 were studied in parallel. The inhibitory effect of GPx2 on NF-κB signaling and its target gene expression was stronger than that of GPx1, whereas cyclooxygenase (COX)- and lipoxygenase (LOX)-derived lipid mediator levels increased more strongly in GPx1 kd than in GPx2 kd cells. Under unstimulated conditions, the levels of the COX-derived prostaglandins PGE_2_ and PGD_2_ were enhanced in GPx2 as well as in GPx1 kd compared to control cells. Specifically, in GPx1 kd cells IL-1β stimulation led to a dramatic shift of the PGE_2_/PGD_2_ ratio towards pro-inflammatory PGE_2_.

Taken together, GPx2 and GPx1 have overlapping functions in controlling inflammatory lipid mediator synthesis and, most probably, exert their anti-inflammatory effects by preventing excessive PGE_2_ production. In view of the high activity of COX and LOX pathways during inflammatory bowel disease our data therefore provide new insights into the mechanisms of the protective function of GPx1 and GPx2 during colitis as well as inflammation-driven carcinogenesis.

## Highlights

• Loss of GPx2 results in higher basal and IL-1β-induced NF-κB activation.
• Suppressive effects of GPx2 on NF-κB are mediated in a redox-dependent manner.
• Both GPx isoforms modulate the lipid mediator profile in response to IL-1β.
• COX-derived prostaglandins increase more strongly in GPx1 than in GPx2 kd cells.

## Graphical abstract

**Figure:**
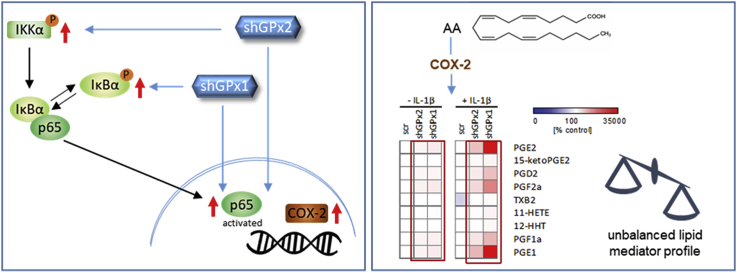

## 1 Introduction

The family of glutathione peroxidases (GPx) is crucial for the regulation of the intra- and extracellular redox balance by reducing hydroperoxides at the expense of glutathione [1]. In humans, there are eight members of this family and five of them (GPx1-4 and GPx6) are selenoproteins having a selenocysteine as part of their catalytic tetrade instead of a cysteine. GPx6 is restricted to the olfactory system, while GPx3 is an extracellular protein which is secreted by epithelial cells and thus most likely contributes to the extracellular antioxidant defense. GPx1, GPx2, and GPx4 are localized inside the cell. Among those, GPx4 is most important because its complete knockout (KO) is embryonically lethal for mice [2] while GPx1 or GPx2 KO mice only have very mild phenotypes [3,4]. One reason for this strong dependency is the fact that GPx4 is the only isoform which has the ability to reduce complex hydroperoxides like phosphatidylcholine hydroperoxide [5]. For GPx1 and GPx2, no specific substrates have been identified so far, but both are able to reduce H_2_O_2_ and fatty acid-derived hydroperoxides. However, they clearly differ in their localization, e.g., along the crypt-villus axis of the intestinal epithelium. Whereas GPx2 is mainly expressed at the crypt bottom, GPx1 and GPx4 are only weakly expressed at this site but mainly found at the luminal surface [6,7]. This distribution pattern suggests a specific function of GPx2 in the homeostatic regeneration of the intestinal epithelium which takes place at the crypt base [6,8]. GPx1 and GPx2 do not only differ with regard to their location but also in the so-called hierarchy of selenoproteins [9,10]. Whereas GPx2 ranks high in hierarchy and is expressed even under selenium restriction, GPx1 expression is highly dependent on the selenium supply indicating that GPx1 is more dispensable.

Under basal conditions, the intestinal epithelium of GPx2 KO mice is working normally, at first glance [4]. Upon loss of GPx2, GPx1 expression is significantly increased at the crypt bottom, whereas GPx4 activity is not affected, indicating that GPx1 strives to compensate the loss of GPx2, at least to some extend [6]. At the same time, GPx2 KO mice develop aberrant apoptotic cells at the crypt base and show more intraepithelial inflammatory cells in comparison to wild type (WT) mice [8]. In GPx1-GPx2 double KO animals, only the re-introduction of GPx2 but not of GPx1 was able to rescue the phenotype of spontaneous ileocolitis and subsequent intestinal cancer formation [[11], [12], [13]]. This was particularly observed under housing conditions which were not specific pathogen-free, indicating a function of GPx2 in preventing inflammation and maintaining intestinal integrity [11]. Indeed, GPx2 has been shown to be upregulated during intestinal inflammation, e.g., during the regeneration of the crypt epithelium after radiation-induced injury [4] and during colitis both in humans [14] and in mice [15]. In the murine dextran sodium sulfate (DSS)-induced colitis model, GPx2 was specifically increased in cells near ulcerations which are involved in the regeneration of the epithelial layer. In the same experiment, GPx1 was only very marginally upregulated during the acute inflammatory phase [15]. Overall, this indicates that there appear to be distinct functions of the two GPx isoforms, and loss of GPx2 in this case cannot be fully compensated by upregulating GPx1. Based on these findings, we aimed to further explore the role of GPx2 in inflammation-related pathways. One obvious candidate pathway is nuclear factor 'κ-light-chain-enhancer’ of activated B-cells (NF-κB) signaling, which is already known to be modulated by GPx1 and GPx4 [16,17].

One of the most important functions of the NF-κB pathway is the activation of the innate immune response. NF-κB signaling is initiated by inflammatory cytokines such as interleukin (IL)-1β and tumor necrosis factor (TNF)-α via their respective receptors as well as by ligands of toll-like receptors and certain growth factor receptor tyrosine kinases (overview in Ref. [18]). NF-κB was the first mammalian transcription factor shown to be redox regulated [19]. NF-κB target genes include, beside others, pro-inflammatory cytokines such as TNF-α, IL-1β, and IL-6 along with enzymes that produce secondary inflammatory mediators such as cyclooxygenase (COX)-2 and inducible NO synthase (iNOS, NOS2). Under resting conditions, the NF-κB dimer (in the canonical pathway usually p50 and p65) is bound in a cytosolic complex with the inhibitor of NF-κB (IκBα). To allow nuclear import and gene activation upon cell stimulation, IκBα needs to be ubiquitinated and degraded by the 26S proteasome. This is typically the consequence of a cascade of phosphorylation events resulting in IκBα phosphorylation by the IKK (IκB kinase) complex [18]. We have shown previously that a stable GPx2 knockdown (kd) in human HT-29 cells results in a strong upregulation of COX-2 in response to loss of GPx2 [14]. COX-2-derived prostanoids are part of the highly orchestrated lipid mediator (LM) network which is responsible for regulating homeostatic as well as inflammatory processes and which needs to be tightly controlled [20].

In this study, we extended the analysis of NF-κB target genes expressed upon loss of GPx2 under basal and IL-1β-stimulated conditions and identified NF-κB as responsible upstream pathway. Next, we directly compared GPx2 and GPx1 kd cells to identify differences between the two isoforms. Based on our results we conclude that both GPx isoforms have an overlapping but also partly distinct function in limiting excessive inflammatory responses via modulation of the cellular redox status.

## 2 Material and methods

### 2.1 Animal experiment

Animal experiments have been approved by the ethics committee of the Ministry of Agriculture and Environment (State Brandenburg, Germany) and all methods were carried out in accordance to permission number V3-2437-29-2012. Male C57BL/6J WT and GPx2-KO [6] mice were housed in individually ventilated cages under specific pathogen-free conditions with a 12-h light/dark cycle and free access to food and water. Mice were weaned onto a diet based on torula yeast (Altromin C1045; Lage, Germany) which was enriched with *l*(+)-selenomethionine (Fisher Scientific, Schwerte, Germany) to a final selenium content of 0.15 mg/kg. Mice were anesthetized with isoflurane (Abbot, Wiesbaden, Germany), and blood was withdrawn by heart puncture. Plasma was obtained after centrifugation for 15 min (1200×*g*, 4 °C). Small intestine and colon were collected and frozen in liquid nitrogen. To obtain protein lysates, frozen tissue samples were homogenized in Tris buffer (100 mM Tris pH 7.6, 300 mM KCl with 0.1% Triton X-100 (Serva, Heidelberg, Germany)) using a TissueLyser (Qiagen, Hilden, Germany) for 2 × 30 s at 30 Hz. Cellular debris was removed by centrifugation (14,000×*g*, 15 min, 4 °C) and protein concentrations were determined by Bradford analysis (Biorad, München, Germany).

### 2.2 Cell culture

HT-29 cells (human colon adenocarcinoma cells; ACC 299 German Collection of Microorganisms and Cell Cultures) were grown in DMEM (high glucose) with 1% nonessential amino acids, 10% FCS (Sigma-Aldrich, Steinheim, Germany), 100 units/mL penicillin, and 100 μg/mL streptomycin (Fisher Scientific) at 37 °C in a 5% CO_2_ atmosphere. To adjust the selenium status, cells were generally grown in the presence of 50 nM sodium selenite (Sigma-Aldrich) for 3 d before starting the experiment. Cells were stimulated with serum-free medium containing 1 ng/mL human recombinant IL-1β (GBF, Braunschweig, Germany) diluted in sterile double-distilled water with or without pretreatment with 20 μM 2-[(aminocarbonyl)amino]-5-(4-fluorophenyl)-3-thiophene carboxamide (TPCA, R & D systems, Minneapolis, USA) or 50 mM N-acetyl cysteine (NAC, Sigma-Aldrich). Cells stably transfected with shRNA against GPx2 or GPx1 or scramble (scr) shRNA were selected with geneticin (Merck, Darmstadt, Germany) or puromycin (Fisher Scientific) when transfected with an expression vector for GFP-GPx2. For lipid mediator analysis, medium supernatants were collected and transferred to solid phase extraction.

### 2.3 Generation of stable cell lines

Generation of shGPx2 and scr control clones has previously been described [14]. Briefly, two different oligonucleotides were selected for the kd of GPx2 and one for GPx1 (Table 1) using the software “siRNA Wizard™ v2.4” (InvivoGen, Toulouse, France). The plasmid psiRNA-h7SKnScr (InvivoGen) containing a scr sequence served as control. The generation of a PLV-lentiviral vector with shRNA-insensitive (non-targetable) GFP-GPx2 has been described previously [21]. The GFP-GPx2 U40S sequence was constructed using the QuikChange Mutagenesis Kit (Stratagene, Amsterdam, Netherlands) according to the manufacturer's instructions and using the primers listed in Table 1. The inducible shRNA against GPx2 and GPx1 (ind_shGPx2#2 and ind_shGPx1#1) was purchased as ready to use plasmid pLKO-puro-IPTG 3xLacO vector (Sigma-Aldrich; Table 1). The lentiviral transfection of HT-29 cells was performed at the UMC Utrecht. Cellular shRNA expression was induced by treating the cells with 1 mM isopropyl-β-*d*-thiogalactopyranoside (IPTG) for three days, and medium was refreshed every other day.

**Table 1:** Sequences used to generate vector constructs.

shGPx2#1	fwd: 5′-ACCTCGATCCTGAACAGTCTCAAGTATCAAGAGTACTTGAGACTGTTCAGGATCTT-3′
	rev: 5′-CAAAAAGATCCTGAACAGTCTCAAGTACTCTTGATACTTGAGACTGTTCAGGATCG-3′
shGPx2#2	fwd: 5′-ACCTCGCTCAACACACAGATCTCCTATCAAGAGTAGGAGATCTGTGTGTTGAGCTT-3′
	rev: 5′-CAAAAAGCTCAACACACAGATCTCCTACTCTTGATAGGAGATCTGTGGTTGAGCG-3′
shGPx1#1	fwd: 5‘-ACCTCGGTACTACTTATCGAGAATGTTCAAGAGACATTCTCGATAAGTAGTACCTT-3
	rev: 5‘-CAAAAAGGTACTACTTATCGAGAATGTCTCTTGAACATTCTCGATAAGTAGTACCG-3‘
ind_sh GPx2 #2	5’-CCGGTGAATGGGCAGAACGAGCATCCTCGAGGATGCTCGTTCTGCCCATTCATTTTTTG-3′
ind_sh GPx1 #1	5′-CCGGCTTCGAGAAGTGCGAGGTGAACTCGAGTTCACCTCGCACTTCTCGAAGTTTTTG-3′
GPx2 U40S	fwd: 5′-GATTGAGAATGTGGCTTCGCTCTCAGGCACAACCACCCGGGACTTC-3′
	rev: 5′-GAAGTCCCGGGTGGTTGTGCCTGAGAGCGAAGCCACATTCTCAATC-3′

### 2.4 Quantitative real-time-PCR

The mRNA was isolated using the Dynabeads mRNA DIRECT Kit (Fisher Scientific) according to the manufacturer's protocol. For cell culture experiments, cells were grown in 12-well plates for 3 d before harvesting. In the mouse study, mRNA was isolated from homogenized frozen intestine. Using 150 fmol oligo (dT) 15 primers and 180 U Moloney Murine Leukemia Virus Reverse Transcriptase (M-MLV RT; Promega, Mannheim, Germany), 150 ng mRNA from cells or 100 ng mRNA from the intestine were transcribed into cDNA by reverse transcriptase PCR. Real-time PCR was performed in a total volume of 25 μL with 1 μL of diluted cDNA and SYBR Green 1 (Molecular Probes, Eugene, USA) as fluorescent reporter using a Mx3005P™ qPCR system (Stratagene). Standard curves from diluted PCR products were used for quantification. cDNA-specific primers (Table 2, Sigma-Aldrich) were designed with Perl Primer v1.1.14 [22]. For cells, RPL13A and OAZ1 were used as reference genes, and for the murine intestinal samples Rpl13a and Epcam were selected.

**Table 2:** Primer sequences (5′→3’).

Gene	Accession number	species	sequence 5’ → 3′
ALOX5	NM_000698.3	human	fwd: GCTGCAACCCTGTGTTGATCC
			rev: AAATGTTCCCTTGCTGGACCTC
ALOX15	NM_001140.3	human	fwd: TGGAGCCTTCCTAACCTACAG
			rev: TCCACATACCGATAGATGATTTCC
COX2	NM_004878.3	human	fwd: ACGCTGCTGGTCATCAAGATG
			rev: TGGCAAAGGCCTTCTTCCGC
Epcam	NM_008532.2	murine	fwd: TCATCGCTGTCATTGTGGTGGT
			rev: TCACCCATCTCCTTTATCTCAGCC
GPX1	NM_000581.2	human	fwd: TACTTATCGAGAATGTGGCGTCCC
			rev: TTGGCGTTCTCCTGATGCCC
GPX2	X68314	human	fwd: GTGCTGATTGAGAATGTGGC
			rev: AGGATGCTCGTTCTGCCCA
Nos2	D26525	human/murine	fwd: CAGCGCTACAACATCCTGGAGG
			rev: GGACCAGCCAAATCCAGTCTGC
OAZ1	NM_004152.2	human	fwd: GCAGCGGATCCTCAATAGCCA
			rev: AGACCCTGGAACTCTCACTGCT
RPL13A	NM_012423.2	human	fwd: AGCCTACAAGAAAGTTTGCCTATCTG
			rev: TAGTGGATCTTGGCTTTCTCTTTCCT
Rpl13a	NM_009438.5	murine	fwd: GTTCGGCTGAAGCCTACCAG
			rev: TTCCGTAACCTCAAGATCTGCT
TNFA	NM_000594.2	human	fwd: GCCTCTTCTCCTTCCTGATCGT
			rev: TGAGGGTTTGCTACAACATGGG
Tnfa	NM_013693	murine	fwd: CCACGTCGTAGCAAACCACC
			rev: TACAACCCATCGGCTGGCAC

### 2.5 Western blot

Whole cell lysates of HT-29 cells were obtained by incubation with RIPA buffer (50 mM Tris pH 7.8, 150 mM NaCl, 2 mM EGTA, 0.1% SDS, 0.5% sodium deoxycholate, and 1% Nonidet P-40) for 2 min at 500 rpm on a plate shaker. Debris was removed by centrifugation (13,000×*g*, 15 min, 4 °C), and protein concentration of the supernatant was determined by Bradford analysis (Biorad). After SDS polyacrylamide gel electrophoresis, gels were immunoblotted to nitrocellulose (2 h, 1.2 mA/cm^2^, 4 °C), and blots were blocked in 5% nonfat dry milk in Tris-buffered saline containing 0.1% Tween 20 at room temperature for 1 h. The following primary antibodies were used to detect proteins: rabbit anti-human GPx2 (GBF, [23]), GPx1 (Abcam #ab108427, Berlin, Germany), GPx4 (Abcam #ab125066), IκBα (Cell signaling #4814, Frankfurt am Main, Germany), phospho-IκBα (Cell signaling #2859), phospho-IKKα (Ser176)/IKKβ (Ser177) (Cell signaling #2078), COX-2 (Cell signaling #12282), Nox1 (Sigma-Aldrich #SAB4200097), GFP (Roche #11814460001, Mannheim, Germany), β-actin (Abcam #ab8227). Horseradish peroxidase conjugated goat-anti-rabbit antibody (Rockland, Limerick, USA) or horse-anti-mouse (Cell signaling #7076) served as secondary antibody. Proteins were detected by chemiluminescence imaging using Supersignal West Dura (Perbio, Bonn, Germany) with the Fuji LAS1000-CCD camera system. Band intensities were quantified densitometrically using the Luminescent Image Analyser LAS-3000 system (Fujifilm, Tokyo, Japan). Protein expression was either normalized to Coomassie blue gel staining or to β-actin, as indicated.

### 2.6 TNF-α ELISA

TNF-α protein levels were measured in plasma samples from mice and in the cell culture supernatants according to the manufacturer's protocol using the mouse TNF-α ELISA MAX Standard Kit and human TNF-α ELISA MAX Standard Kit (BioLegend, London, UK), respectively. 100 μL undiluted plasma samples were used. The cell culture supernatant was diluted 1:1000.

### 2.7 Glutathione peroxidase activity

Cells were lysed in homogenization buffer (100 mM Tris pH 7.6, 300 mM KCl, 0.1% Triton X-100), sonicated, and centrifuged (20,000×*g*, 10 min, 4 °C). Total GPx activity was measured by the glutathione reductase-coupled test using 96-well plates [24]. After 10 min preincubation at 37 °C with all the assay components, the reaction was started by the addition of different substrates. Herein, H_2_O_2_ (Sigma-Aldrich), hydroxy-octadecadienoic acid (HPODE), hydroxy-eicosatetraenoic acid (HPETE) or tert-butyl hydroperoxide (TBHP, Sigma-Aldrich) were used to reach a final concentration of 50 μM. The reaction was measured for 2 min at 340 nm using a microplate absorbance reader (Biotech Instruments, Bad Friedrichshall, Germany). According to Lambert–Beer's law, one unit generally is defined as consumption of 1 μmol NADPH/min and expressed as mU/mg protein.

### 2.8 NF-κB reporter gene assay

The NFkB reporter gene plasmid (3× κB-Luc) was supplied by Addgene (Plasmid 26699, [25]). The Renilla luciferase plasmid (Promega) was co-transfected to normalize for differences in transfection efficiency. Using 500 μL DMEM without antibiotics, 2 × 10^5^ HT-29 cells were seeded onto 24-well plates. After 24 h, cells were transfected with 0.5 μg 3× κB-Luc reporter plasmid and 0.05 μg Renilla luciferase plasmid diluted in Opti-MEM (Fisher Scientific) using Lipofectamin (Fisher Scientific) according to the manufacturer's protocol. 24 h after transfection, cells were either stimulated with IL-1β or received fresh serum-free medium. After 24 h, cells were lysed in 150 μL of reporter lysis buffer (Promega). 10 μL of the lysate was used to measure firefly luciferase activity by adding 100 μL dissolved luciferin, and 20 μL lysate were used to analyzed Renilla luciferase activity by adding 75 μL renilla substrate (Renilla luciferase assay system, Promega) on a Luminoskan Ascent (Labsystems, Finland).

### 2.9 Solid phase extraction of lipid mediators

LMs were extracted from cell culture supernatants as described [26]. In brief, supernatants were transferred to ice-cold methanol (supernatant/methanol = 40/60) containing deuterium-labeled internal standards (200 nM d_8_-5S-HETE, d_4_-LTB_4_, d_5_-LXA_4_, d_5_-RvD2, d_4_-PGE_2_, and 10 μM d_8_-AA; Cayman Chemical/Biomol, Hamburg, Germany). After protein precipitation at −20 °C, samples were centrifuged, acidified (pH 3.5) and subjected to solid phase extraction (Sep-Pak® Vac 6 cc 500 mg/6 mL C18; Waters, Milford, USA). The cartridges were successively washed with methanol and *n*-hexane, and LMs were eluted with methyl formiate. Eluates were evaporated to dryness (TurboVap LV, Biotage, Uppsala, Sweden) and LMs taken up in methanol/water (50/50) for UPLC-MS/MS analysis.

### 2.10 Lipid mediator analysis by UPLC-MS/MS

LMs were separated on an ACQUITY UPLC® BEH C18 column (1.7 μm, 2.1 × 100 mm; Waters, Eschborn, Germany) at 50 °C using an Acquity™ UPLC system (Waters) as previously described [26]. Briefly, the eluent methanol-water-acetic acid was ramped at 0.3 mL/min from 42:58:0.01 to 86:14:0.01 over 12.5 min and then to 98:2:0.01 for 3 min. The LC system was coupled to a QTRAP 5500 ESI tandem mass spectrometer (Sciex, Darmstadt, Germany), which was operated in the negative ionization mode using scheduled multiple reaction monitoring. Parameters were adjusted as reported [26]. The retention time and at least six diagnostic ions for each LM were confirmed using an external standard (Cayman Chemical/Biomol). Quantification was achieved by calibration curves for each LM as described [26].

### 2.11 Determination of the cellular redox status

Dihydrorhodamin (DHR123) was used as fluorescent dye. Cells were incubated for 45 min with 20 μM DHR123 at 37 °C and after a washing step, they were stimulated for 60 min with 1 mM H_2_O_2_, 50 μM TBHP, 50 μM HPODE or 50 μM HPETE in phenol-red-free and serum-free RPMI 1640 medium at 37 °C. Accordingly, rhodamin fluorescence was measured using a microplate absorbance reader (Biotech Instruments) after discarding the medium and washing the cell layer. Values were normalized to viable cells analyzed by neutral red assay which was performed afterwards.

### 2.12 Statistics

Values are presented as mean + SD. The Student's *t*-test (unpaired, two-tailed) was performed to analyze two groups, one-way ANOVA for comparing more than two groups, and two-way ANOVA for two-parametric data with Bonferroni's post-hoc test using Graphpad Prism 6. Differences with a p value of less than 0.05 were considered statistically significant.

## 3 Results

### 3.1 NF-kB target genes are induced upon loss of GPx2

**Fig. 1:**
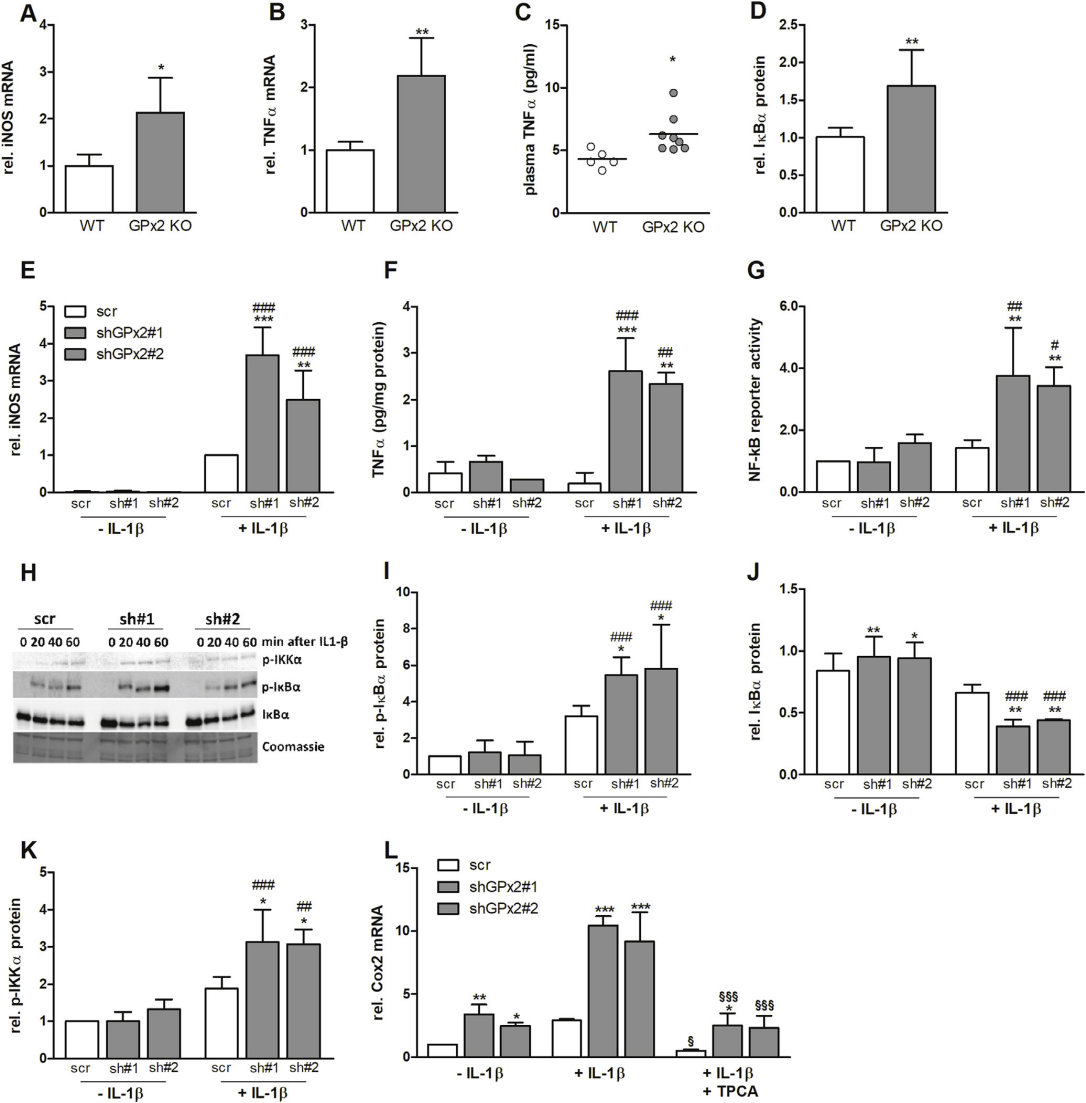
**Deletion of GPx2 enhances inflammatory protein expression and NF-κB activity in vivo and in vitro**. In the intestine of WT and GPx2 knockout (GPx2 KO) mice, mRNA levels of iNOS (A) and TNF-α (B) were analyzed by qPCR and normalized to Rpl13a and Epcam. Plasma levels of TNF-α were analyzed by ELISA (C) and intestinal protein levels of IκBα were analyzed by Western blot and normalized to β-actin (D). In HT-29 cells, stable kds were generated using two different shRNA constructs against GPx2 (sh#1 and sh#2) and compared to cells expressing a non-targeting shRNA sequence (scr). All cells were supplemented with 50 nM sodium selenite for 72 h and stimulated with 1 ng/mL IL-1β. After 3 h of IL-1β stimulation, mRNA levels of iNOS (E) were measured by qPCR and secretion of TNF-α into the medium was measured by ELISA after 4 h of IL-1β stimulation (F). An NF-κB reporter gene assay was performed with and without IL-1β stimulation for 24 h and normalization to Renilla luciferase activity for transfection efficiency (G). Protein levels of IκBα, p-IκBα, and p-IKKα were analyzed at different time points as indicated (H). Quantification was performed at 40 min after IL-1β stimulation for IκBα and p-IκBα (I and J) and after 20 min for p-IKKα (K). COX-2 mRNA was analyzed after 3 h of IL-1β stimulation, with or without pretreatment with 20 μM TPCA for 1 h (L). Data are given as means + SD (n = 3). *p < 0.05; **p < 0.01; ***p < 0.001 vs. respective WT or scr control, ^#^p < 0.05; ^##^p < 0.01; ^###^p < 0.001 vs. respective unstimulated control (IL-1β); and ^§^p < 0.05; ^§§§^p < 0.001 vs. +IL-1β analyzed by two-way ANOVA with Bonferroni's post-test.

GPx2 KO mice are characterized by low-grade intestinal inflammation [8]. Therefore, we analyzed the mRNA expression of the pro-inflammatory mediator TNF-α and of the enzymes iNOS and COX-2 in the intestine of WT and GPx2 KO mice which were fed a selenium-adequate diet. mRNA levels of iNOS and TNF-α were upregulated in the intestine of GPx2 KO mice (Fig. 1A and B) while COX-2 only showed a trend (data not shown). TNF-α levels were also increased in the plasma of GPx2 KO in comparison to WT mice (Fig. 1C). Interestingly, also the levels of IκBα were elevated in the intestinal epithelium of GPx2 KO mice (Fig. 1D), suggesting that IκBα is upregulated in a compensatory manner to reduce NF-κB activation in GPx2 KO animals.

To investigate the underlying signaling cascades, the human colorectal cancer cell line HT-29 was chosen as model. We have previously shown that COX-2 is strongly upregulated in HT-29 cells with a stable shRNA-mediated kd of GPx2 [14]. This was detectable both under basal as well as under IL-1β-stimulated conditions using single cell clones transfected with two independent shRNA constructs against GPx2. Similar to COX-2 expression, cells with GPx2 kd also expressed increased level of iNOS mRNA (Fig. 1E) and secreted more TNF-α into the medium as compared to scr control cells (Fig. 1F). Both effects were only evident after stimulation with 1 ng/mL IL-1β for 3 or 4 h, respectively. All three proteins, COX-2, iNOS, and TNF-α, are known to be regulated via the transcription factor NF-κB [18]. NF-κB as well as upstream kinases and phosphatases are redox-sensitive and might, therefore, be modulated by GPx2. Using a reporter gene assay, no differences in basal NF-κB activity were detected whereas in IL-1β-stimulated cells the luciferase activity was higher in GPx2 kd than in scr cells (Fig. 1G). Similar to the *in vivo* results, IκBα was slightly but significantly upregulated in GPx2 kd cells under unstimulated conditions (Fig. 1J). Upon IL-1β stimulation, IκBα phosphorylation increased, and this increase was more pronounced in GPx2 kd cells than in scr cells (Fig. 1H and I). Accordingly, IκBα levels decreased more strongly in GPx2 kd cells (Fig. 1J) which is supposed to result from its enhanced degradation. IκBα becomes sensitive for degradation via phosphorylation by the upstream kinases IKKα and IKKβ. Indeed, phosphorylation and thereby activation of IKKα was increased in GPx2 kd compared to scr cells after IL-1β stimulation (Fig. 1K). COX-2 mRNA expression was chosen as exemplarily read-out to show that the activation of NF-κB upon loss of GPx2 could be reversed by co-treatment with the IKKβ inhibitor TPCA (Fig. 1L). In summary, these data suggest that high GPx2 expression appears to inhibit IL-1β-stimulated NF-κB activation, which might involve decreased IKK activity.

### 3.2 Pro-inflammatory effects in GPx2-deficient cells can be rescued by redox-active GPx2

**Fig. 2:**
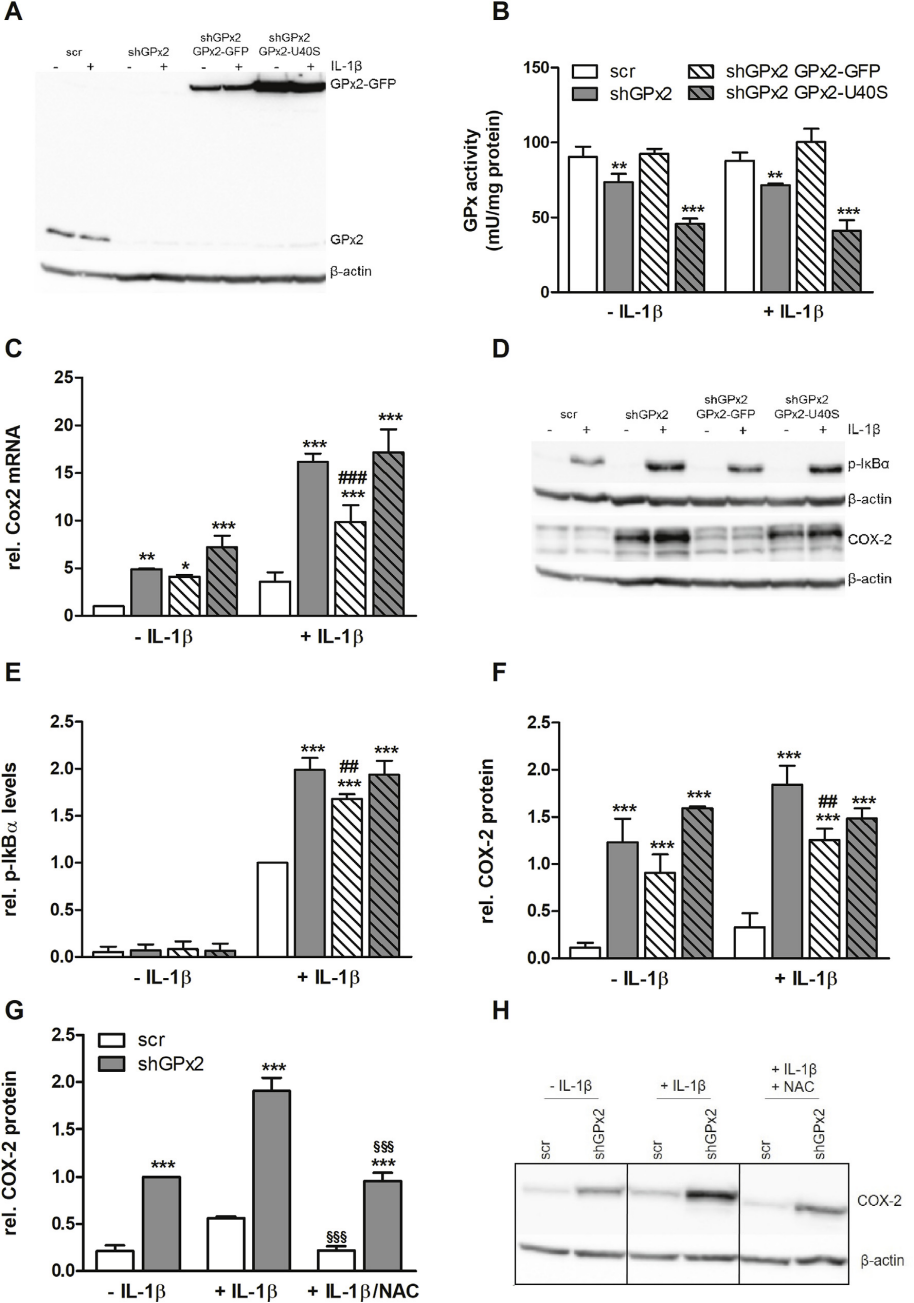
**Suppression of pro-inflammatory signal transduction requires redox-active GPx2**. Stable GPx2 kd HT-29 cells (shGPx2) were further transfected with a non-targetable GFP-tagged GPx2 (shGPx2 GPx2-GFP) or with a GFP-tagged mutant form of GPx2 (shGPx2 GPx2-U40S). After supplementation with 50 nM sodium selenite for 72 h, endogenous GPx2 and GFP-tagged GPx2 expression was analyzed by Western blot 24 h after stimulation with 1 ng/mL IL-1β (A). In addition, GPx activity was spectrophotometrically determined using H_2_O_2_ as substrate (B). Cells were stimulated for 3 h with IL-1β and transcript levels of COX-2 were measured by qPCR and normalized to Rpl13a and Oaz1 (C). In the same lysates used in (A), COX-2 protein levels were detected by Western Blot (D, F). Cells were stimulated for 1 h with IL-1β and p-IκBα protein levels were detected by Western Blot (D, E). Cells were stimulated for 4 h with IL-1β with or without pretreatment for 1 h with 50 mM NAC, and COX-2 protein levels were measured by Western blot (G and H). Western blot bands were normalized to β-actin. Data are given as means + SD (n = 3). *p < 0.05; **p < 0.01; ***p < 0.001 vs. respective scr; ^#^p < 0.05; ^##^p < 0.01; ^###^p < 0.001 vs. shGPx2 and ^§§§^p < 0.001 vs. +IL-1β analyzed by two-way ANOVA with Bonferroni's post-test.

It is well established that an increased oxidative tone results in phosphatase inhibition and thus prolonged phosphorylation of different kinases including IKKs [27]. To better understand the mechanistic basis of the GPx2 effects on the NF-κB pathway, we rescued GPx2 expression in the shGPx2#1 clone either by transfecting a non-targetable GFP-tagged active GPx2 or an inactive mutant with a serine instead of the selenocysteine (GPx2-U40S) (Fig. 2A). As expected and described previously [14], total GPx activity was reduced by about 30% in cells with kd of GPx2 using H_2_O_2_ as substrate (Fig. 2B). This loss of activity was completely rescued by introducing redox-active GPx2 while the inactive U40S mutant reduced total GPx activity even further in comparison to scr cells (Fig. 2B). This kind of overcompensation has also been described for an inactive mutant of GPx4 [28], but might also result from higher expression of GPx2-U40S in comparison to GPx2-GFP (Fig. 2A). The increased level of COX-2 in cells with GPx2 kd after IL-1β stimulation could be partially restored by re-introducing redox-active GPx2 but not by the inactive mutant U40S on both mRNA and protein level (Fig. 2C, D, F). Similarly, levels of p-IκBα were reduced by GPx2-GFP in GPx2 kd cells, whereas the inactive GPx2-U40S mutant failed in this respect (Fig. 2E, D). To further confirm that the GPx2 kd upregulates COX-2 expression in a redox-dependent manner, we co-stimulated cells with IL-1β and NAC, a precursor of cellular glutathione, which resulted in lower COX-2 protein levels in comparison to cells treated with only IL-1β (Fig. 2G and H). Based on these findings it can be concluded that GPx2 regulates NF-κB via a redox-regulated process.

### 3.3 Effects on NF-kB activity are also observed upon knockdown of GPx1

**Fig. 3:**
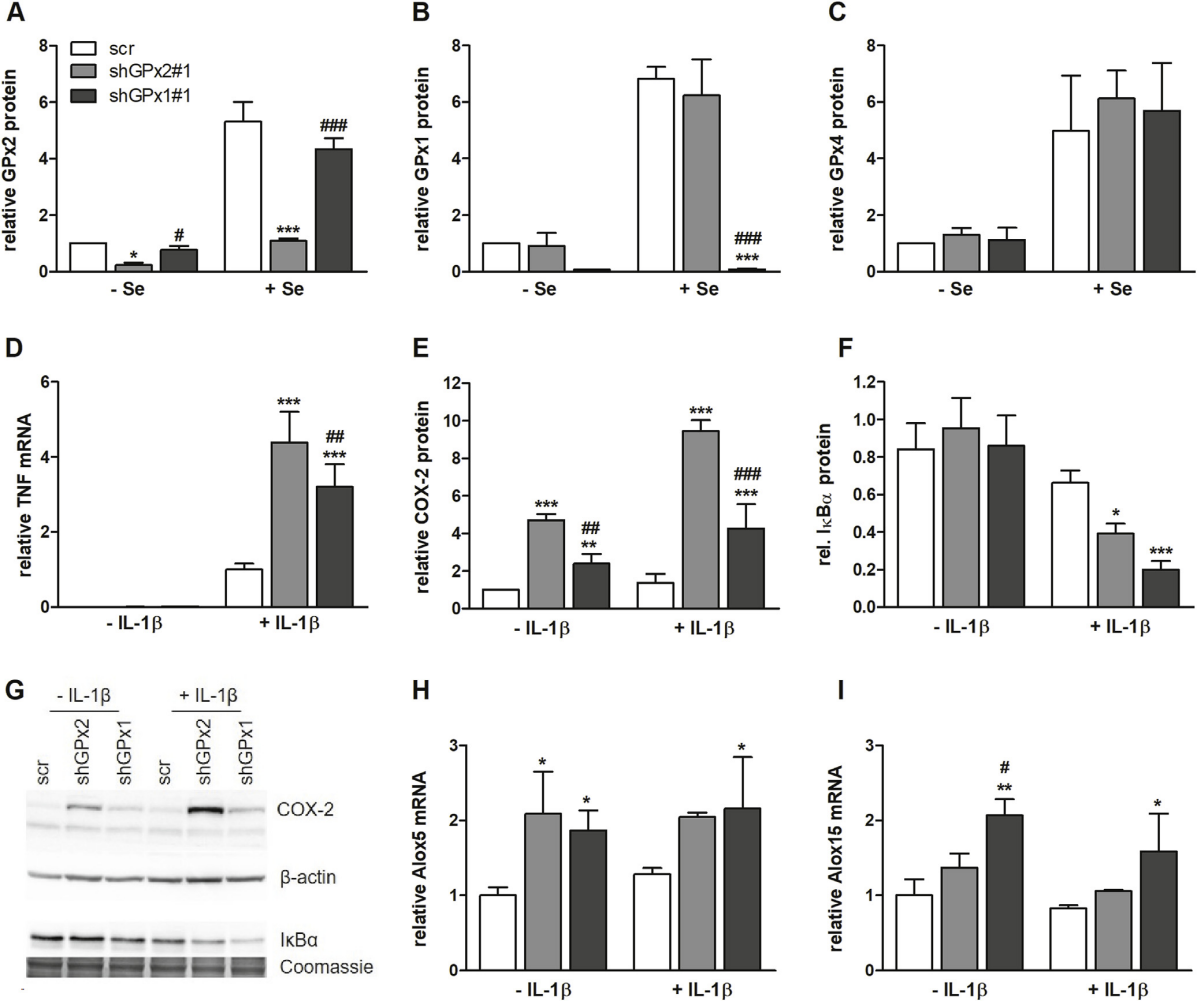
**Comparison of NF-κB activation in GPx2 and GPx1 kd cells**. Scr, GPx1 and GPx2 kd HT-29 cells (shGPx1#1 and shGPx2#1) were supplied without (-Se) or with 50 nM sodium selenite (+Se) for 72 h. Protein expression of GPx2, GPx1, and GPx4 was detected by Western Blot (A–C). Cells were cultured with 50 nM sodium selenite for 72 h and accordingly were stimulated with or without 1 ng/mL IL-1β (D–I). TNF-α mRNA (IL-1β for 3 h, D), COX-2 (IL-1β for 6 h, E), and IκBα (IL-1β for 30 min, F) protein as well as Alox5, and Alox15 mRNA levels (IL-1β for 3 h, H–I) were measured by qPCR and normalized to Rpl13a and Oaz1 or by Western Blot, respectively. Representative Western blots are shown (G) and bands were densitometrically analyzed and normalized to β-actin or Coomassie stains. Data are given as means + SD (n = 3). *p < 0.05; **p < 0.01; ***p < 0.001 vs. scr and ^#^p < 0.05; ^##^p < 0.01; ^###^p < 0.001 vs. shGPx2#1 analyzed by two-way ANOVA with Bonferroni's post-test.

**Fig. S1:**
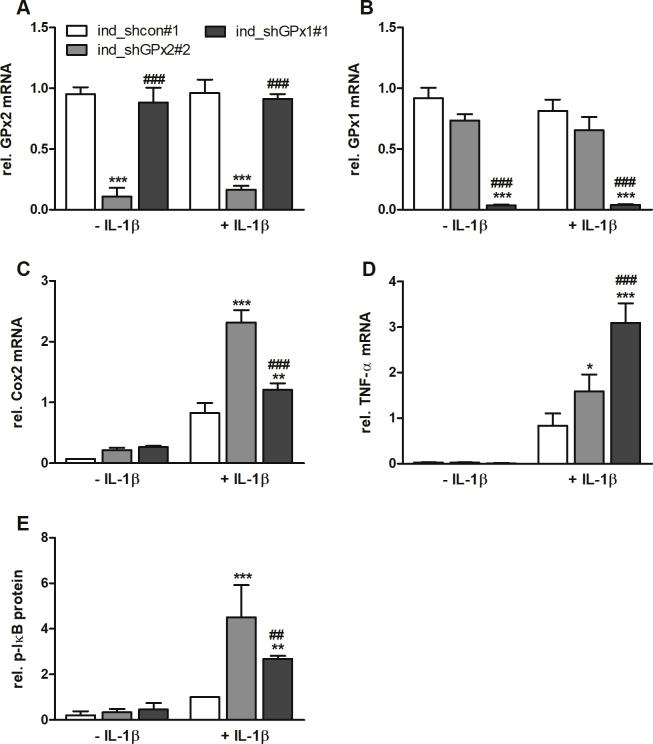
**NF-κB signaling in inducible GPx2 and GPx1 kd cells.** HT-29 cells were stably transfected with an inducible shRNA against GPx2 (ind_shGPx2#2), GPx1 (ind_shGPx1#1) or a non-specific construct (ind_shcon#1). Cells were stimulated with 1 mM IPTG and 50 nM sodium selenite for 72 h and medium was refreshed every day. Afterwards, cells were stimulated for 4 h with 1 ng/mL IL-1β and GPx2 (A), GPx1 (B), COX-2 (C), and TNF-α (D) transcript levels were measured by qPCR and normalized to Rpl13a and Oaz1. pIκBα protein levels were analyzed by Western Blot 4 h after 1 ng/mL IL-1β stimulation and normalized to Coomassie staining (E). Data are given as means + SD (n = 3). *p < 0.05; **p < 0.01; ***p < 0.001 vs. ind_shcon#1 and ^##^p < 0.01; ^###^p < 0.001 vs. ind_shGPx2#2 analyzed by two-way ANOVA with Bonferroni's post-test.

Redox-regulatory properties have also been described for other members of the GPx family. Thus, we aimed to directly compare effects of suppressing either GPx2 or GPx1 expression by kd in HT-29 cells. The GPx1 kd efficiency was higher than that of GPx2 (Fig. 3A and B). Especially under selenium adequate conditions, GPx1 was almost undetectable in kd cells but highly expressed in scr cells. GPx4, the third intracellular GPx belonging to the selenoprotein family, was unaffected by kd of either GPx1 or GPx2 (Fig. 3C). When directly comparing GPx1- and GPx2-modulated effects on the expression of NF-κB target genes, such as COX-2 and TNF-α, we observed superior upregulation of these proteins in GPx2 as compared to GPx1 kd cells (Fig. 3D, E, G). Investigating upstream pathway components revealed that kd of GPx2 as well as GPx1 decreased the level of IκBα (Fig. 3F and G). To exclude long-term compensatory effects during stable kd, which might have modulated the cellular redox status, we used vectors for IPTG-inducible shRNA against GPx2 or GPx1. Using this different approach, we could essentially confirm the results described above (Fig. S1Fig. S1). Differences between both GPx kds were not totally consistent between the constitutive and the inducible system indicating that both isoforms are able to block IL-1β-induced NF-κB activation. In addition to COXs, lipoxygenases (LOX) are crucial enzymes for LM biosynthesis. 5-LOX (ALOX5) mRNA expression was slightly increased in both, GPx2 and GPx1 kd cells (Fig. 3H) whereas 15-LOX-1 (ALOX15) was only substantially upregulated upon kd of GPx1 (Fig. 3I).

### 3.4 Knockdowns of GPx2 and GPx1 shift the prostaglandin profile towards pro-inflammatory PGE_2_

**Fig. 4:**
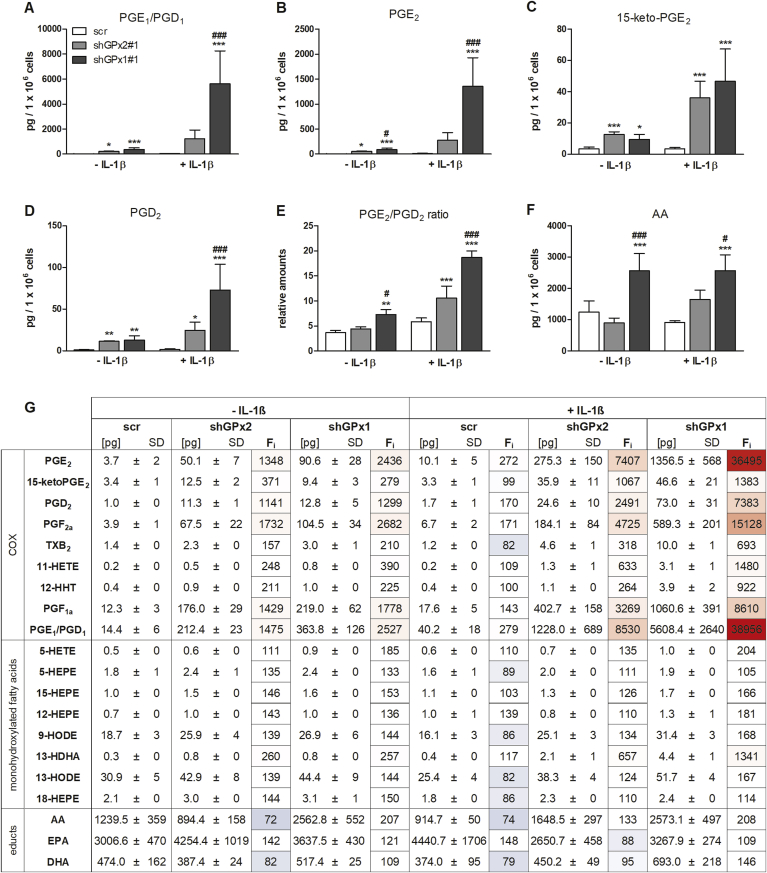
**Effect of GPx2 and GPx1 kd on lipid mediator profiles**. Scr, GPx1 and GPx2 kd HT-29 cells were supplemented with 50 nM sodium selenite for 72 h. Cells were stimulated for 24 h with 1 ng/mL IL-1β, and LM profiles were analyzed by UPLC-MS/MS. Levels of selected LMs and educts are shown (A–F) together with a heatmap for the whole LM profile (G). The values and color scale of the F_i_ column refers to changes in the percentage of LMs vs. scr without IL-1β. The values in the other columns give the concentrations of LMs in pg per 1 × 10^6^ cells. Data are given as means + SD (n = 4). *p < 0.05; **p < 0.01; ***p < 0.001 vs. scr and ^#^p < 0.05; ^##^p < 0.01; ^###^p < 0.001 vs. shGPx2#1 analyzed by two-way ANOVA with Bonferroni's post-test. To compare basal levels of LMs (without IL-1β treatment), one-way ANOVA with Bonferroni's post-test was applied. HHT hydroxy-heptadecatrienoic acid, HETE hydroxy-eicosatetraenoic acid, HODE hydroxy-octadecadienoic acid, HEPE hydroxy-eicosapentaenoic acid, HDHA hydroxy-docosahexaenoic acid, AA arachidonic acid, EPA eicosapentaenoic acid, DHA docosapentaenoic acid. (For interpretation of the references to color in this figure legend, the reader is referred to the Web version of this article.)

COX-2 and the LOX isoenzymes participate in the biosynthesis of potent pro- and anti-inflammatory LMs, i.e., prostaglandins (PG), leukotrienes and specialized pro-resolving LMs. Whether this interconnected LM network is regulated by GPx1 and GPx2 was investigated using a metabolipidomics approach. Both kd of GPx2 and GPx1 strongly increased the formation of COX-derived prostanoids under IL-1β-stimulated as well as unstimulated conditions with more pronounced effects in GPx1 kd than in GPx2 kd cells (Fig. 4A, B, D, G). The higher levels of COX products, including also HETEs and HDHAs, in GPx1 vs. GPx2 kd cells (Fig. 4G) cannot be explained from higher COX-2 expression, which was actually lower in GPx1 kd cells (Fig. 3E), but might partially derive from the increased availability of free arachidonic acid (AA, Fig. 4F) and docosahexaenoic acid (DHA, Fig. 4G). In contrast, neither the levels of eicosapentaenoic acid (EPA) nor its oxygenized products, the HEPEs (Fig. 4G) substantially differed between GPx1 and GPx2 kd cells as it was the case for oxygenation products of linoleic acid, 9-HODE and 13-HODE (Fig. 4G).

The preferential increase in prostanoid levels as compared to other LMs by GPx1 kd might further point to an elevated activity of either COX-2 or terminal prostanoid synthases. Remarkably, prostanoids that derive from microsomal prostaglandin E_2_ synthase (mPGES)-1, i.e., PGE_1_ (Fig. 4A) and PGE_2_ (Fig. 4B), were strongest elevated relative to the unstimulated scr control among all LMs analyzed: ~8000 fold in IL-1β-stimulated GPx2 kd and ~37,000 fold in GPx1 kd cells (Fig. 4G). In contrast, the inactive PGE_2_ metabolite 15-keto-PGE_2_ was upregulated in both kd lines to a similar extent, which rather excludes specific effects of GPx isoforms on PGE_2_ degradation (Fig. 4C). The level of COX-derived PGD_2_, which has overall anti-inflammatory activities, was also substantially increased, in particular in GPx1 kd cells upon stimulation with IL-1β (Fig. 4D). Accordingly, also the PGE_2_/PGD_2_ ratio was shifted towards pro-inflammatory prostaglandins which was again more pronounced in GPx1 kd cells (Fig. 4E).

To investigate the influence of LOX isoenzymes on GPx-dependent fatty acid oxygenation, we determined their mRNA expression levels. 15-LOX-1 mRNA levels were relatively low in non-stimulated HT-29 cells (Ct values around 27 for results shown in Fig. 3I) while mRNA levels of 5-LOX were in a comparable range as COX-2 (Ct values of 23 and 20, respectively). Treatment with IL-1β upregulated 5-LOX mRNA expression in both kd cell lines (Fig. 3H). The putative 5-LOX product 5-HETE was increased in GPx1 kd vs. GPx2 kd cells (Fig. 4G), whereas 5-HEPE, another 5-LOX product, was unaffected by the GPx status (Fig. 4G). Whether these mono-hydroxylated fatty acids are produced by 5-LOX or non-enzymatic autooxidation cannot be clearly answered. Along these lines, we could not detect specific 5-LOX products such as the leukotriene (LT)B_4_. 15-LOX-1 mRNA was exclusively upregulated in GPx1 kd cells (Fig. 3I). This effect is not consistent with the regulation of the 15-LOX-1 product 15-HEPE, which was upregulated in both kd lines (Fig. 4G). Together, overall LM biosynthesis was more strongly induced by GPx1 than GPx2 kd, which was accompanied by a marked elevation of pro-inflammatory PGE_2_ formation.

### 3.5 Differences in total GPx activity by loss of GPx2 or GPx1 are not reflected by the cellular redox status

**Fig. 5:**
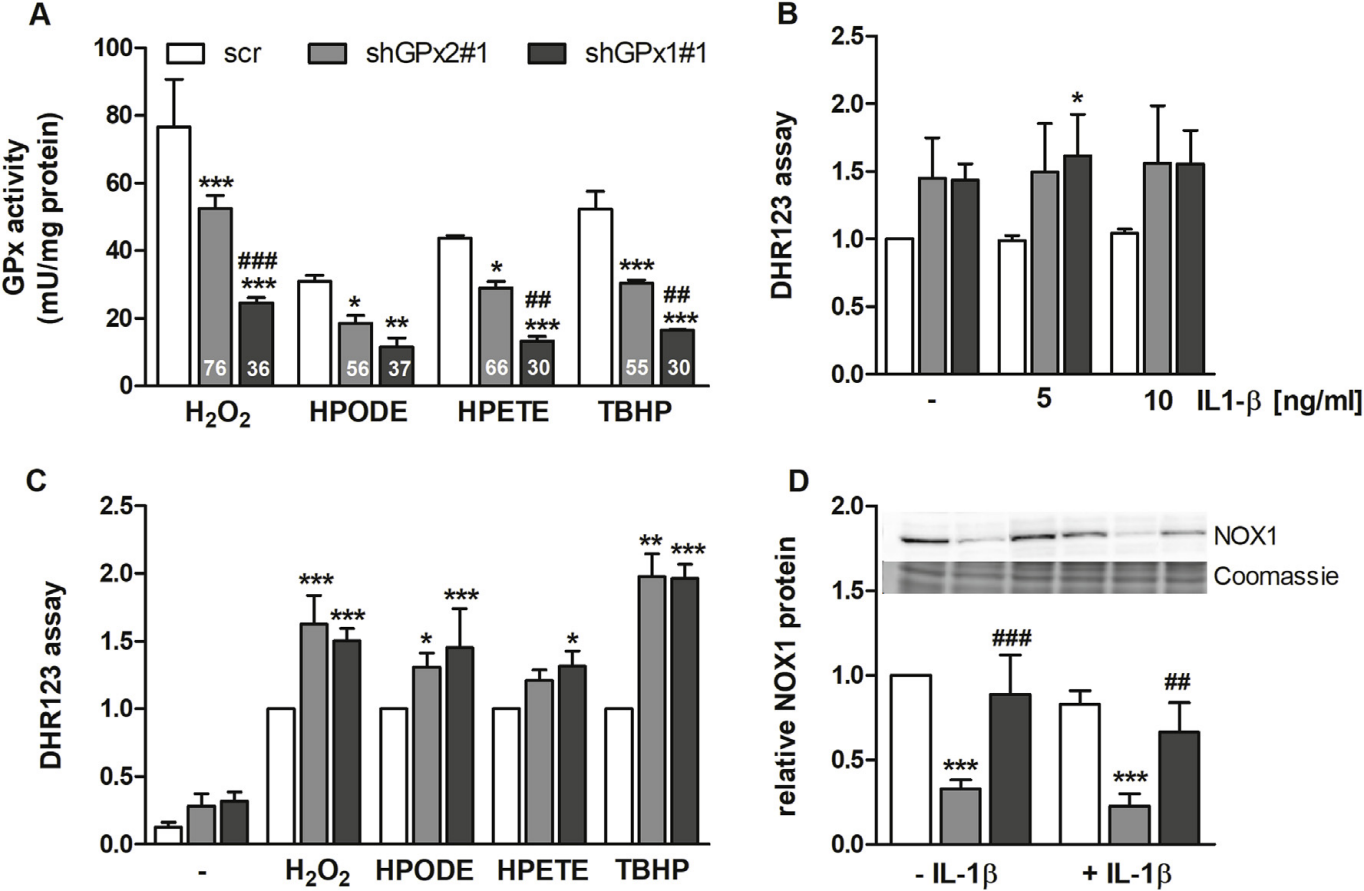
**Redox parameters of GPx2 and GPx1 kd cells**. Scr, GPx1 and GPx2 kd HT-29 cells were supplemented with 50 nM sodium selenite for 72 h. Cells were harvested and GPx activity was measured using different substrates (H_2_O_2_, HPODE, HPETE, and TBHP) with a final concentration of 50 μM (A). Numbers within the bars represent percentages in comparison to the respective scr set as 100%. For the DHR123 assay, cells were incubated with 5 μM DHR for 45 min followed by 1 h treatment with or without 5 or 10 ng/mL IL-1β (B) or 1 mM H_2_O_2_, 50 μM HPODE, HPETE, or TBHP (C). Values of the DHR assay were normalized to viable cells analyzed by neutral red assay. NOX1 protein levels were analyzed by Western Blot 4 h after 1 ng/mL IL-1β stimulation and normalized to Coomassie staining. Data are given as means + SD (n = 3). *p < 0.05; **p < 0.01; ***p < 0.001 vs. scr and ^##^p < 0.01; ^###^p < 0.001 vs. shGPx2#1 analyzed by two-way ANOVA with Bonferroni's post-test.

As described before, the kd of GPx2 reduced the total GPx activity against H_2_O_2_ to 76% of scr-transfected control cells (Fig. 5A and [14]). At the same time, GPx1 kd cells exhibited only 36% of the GPx activity against H_2_O_2_ compared to scr cells. Comparable results were obtained when using the hydroperoxides HPETE and TBHP as substrates in the GPx activity assay. Aside from HPODE as substrate, significant differences between the GPx2 and GPx1 kd cells were observed. This indicates that the cellular GPx activity in HT-29 cells is much stronger impaired by the loss of GPx1 than of GPx2 for a broad spectrum of hydroperoxide substrates.

Next, we analyzed the cellular redox status using DHR123 as fluorescent dye to correlate cellular GPx activity to the redox status in response to the treatment of cells with the different GPx substrates. Basal levels of reactive oxygen species (ROS) were increased to a comparable extent in both GPx kd clones (Fig. 5B and C). Next, we investigated whether redox parameters are changed in response to IL-1β stimulation. IL-1β failed to increase the ROS-derived fluorescence signal, possibly due to the low sensitivity of the assay (Fig. 5B). Treatment with the four hydroperoxides analyzed in the GPx activity assay significantly increased the cellular content of ROS in all cell lines but to a higher extend in the GPx kd than in the scr cells. Again, we could not observe differences between GPx2 and GPx1 kd cells in this respect (Fig. 5C). Thus, GPx activity did not clearly correlate with the cellular redox status. As described for peroxiredoxins, GPxs might not affect the overall cellular redox status that much but could locally modulate hydroperoxide availability, which however might not be detectable by the DHR123 assay.

Because of the increased amounts of endogenous ROS observed in GPx2 and GPx1 kd cells, we analyzed the expression of NADPH oxidase (NOX)1, which is responsible for endogenous H_2_O_2_ production. Intriguingly, NOX1 protein levels were strongly reduced in GPx2 kd cells in comparison to the scr control but remained unaffected in GPx1 kd cells. This effect was independent of IL-1β stimulation (Fig. 5D). This indicates that the high basal ROS levels that are observed in GPx1 kd cells might partially depend on NOX1 while elevated ROS levels of GPx2 kd cells appear to derive from a different source as NOX1 was reduced in parallel to GPx2.

## 4 Discussion

In this study we could show for the first time that GPx2 is able to limit NF-κB signaling both *in vivo* and *in vitro*. This is in line with effects described for other GPx isoforms, especially GPx1 and GPx4. In different model systems, it has been clearly shown that GPxs are important players for balancing NF-κB activity. For example, GPx1 KO mice showed an enhanced inflammatory response in comparison to WT mice during endotoxemia [29]. In addition, GPx1/GPx2 double KO mice spontaneously developed an ileocolitis [30]. In both cases, NF-κB was proposed to be the main pathway mediating those effects. Caco-2 cells with selenium depletion exhibited decreased expression of GPx1 and increased TNFα-stimulated NF-κB reporter gene activity [31]. Inversely, cells overexpressing GPx1 [16] or GPx4 [17] displayed reduced NF-κB activation. In MCF-7 cells, GPx1 overexpression reduced the NF-κB response towards UV irradiation, TNF-α, or H_2_O_2_ treatment [32] which was mediated by suppressing IKKα activity.

Here we show that pro-inflammatory factors such as TNF-α and iNOS, which are known to be regulated via the transcription factor NF-κB [33,34], were increased in GPx2 KO mice both in the intestine and in case of TNF-α also systemically in the plasma (Fig. 1A–D). Previously, we observed higher intraepithelial F4/80 positive cells in the intestinal epithelium which could explain these effects [8]. Based on those observations we chose the human epithelium-derived HT-29 cells as suitable *in vitro* model to study the influence of the pro-inflammatory cytokine IL-1β on downstream NF-κB signaling. We could clearly show that the whole NF-κB signaling cascade was activated in response to IL-1β starting from the phosphorylated IKK complex (Fig. 1K). This activation was stronger in cells with GPx2 kd versus scr control cells. The IL-1β-induced effect could be prevented by pretreating cells with TPCA, an IKKβ inhibitor, indicating that this kinase complex is essentially involved in NF-κB activation in these cells. In addition, the enhanced IL-1β-induced NF-κB activation was dependent on a redox-active GPx2 because IL-1β-induced effects were partially restored only by WT-GPx2 and not by the inactive U40S mutant (Fig. 2C–F). Pretreating cells with NAC only reduced IL-1β-stimulated COX-2 levels and did not abrogate basal differences (Fig. 2G and H). This implicates that the basal increase in COX-2 expression in GPx2 kd cells is not mediated through an NF-κB-dependent COX-2 upregulation due to released or serum-derived IL-1β. Under constitutive conditions, other transcription factors have been shown to modulate COX-2 expression. One of those is NFAT but also others are involved depending on the cell type studied [35]. The strong downregulation of NOX1 in GPx2 kd cells independent of IL-1β treatment (Fig. 5D) might give another explanation. In PMA-treated CHL cells, NOX1 activation has been shown to control COX-2 expression resulting in a reciprocal expression [36], which is in line with our findings.

Next, we aimed to identify specific functions of the two isoforms GPx1 and GPx2 which are undefined so far. The only clear differences described comprise localization and response to limited selenium supply which is called hierarchy of selenoproteins [10]. In addition, GPx2 is regulated via the transcription factor Nrf2 [37] which is, beside others, important to induce the expression of antioxidant enzymes in response to cellular oxidative stress [18]. Thus, it can be anticipated that GPx1 is the constitutively expressed isoform while GPx2 is induced specifically to limit increased amounts of hydroperoxides, and this response then needs to be as independent as possible from the selenium status of the cell. Interestingly, expression of COX-2 protein (Fig. 3E) and TNF-α mRNA levels (Fig. 3D) were significantly increased in cells with GPx2 as well as GPx1 kd indicating overlapping functions of GPx2 and GPx1 in preventing an excessive inflammatory response, even though effects were stronger in GPx2 kd cells. In line with increased COX-2 expression levels, also COX-2 product formation seems to be strongly enhanced in the two GPx kd cell lines reflected by substantial amounts of prostaglandins, especially PGE_1_, PGE_2_ and PGD_2_, both under basal and IL-1β-stimulated conditions (Fig. 4). However, this effect was more pronounced in GPx1 than in GPx2 kd cells. The observed increase in COX-2 product formation could be the result from different partially overlapping effects: i) PGs could derive from an elevated ability to release AA or other educts from the plasma membrane, ii) their formation could be enhanced by higher expression of COX-2 itself and/or downstream PG synthases, iii) their respective enzymatic activity could be enhanced or iv) the degradation of PGs could be impaired. Indeed, AA levels were enhanced more strongly in GPx1 than in GPx2 kd cells (Fig. 4F). In GPx2 kd cells, mPGES-1 mRNA expression was shown to be enhanced [14], which however has not been analyzed for GPx1 kd cells so far. Regarding iii), it is well-established that COX enzymes require hydroperoxides for their activity [38]. Based on that, GPx1 was supposed to inhibit COX activity by lowering the cellular hydroperoxide tone [10]. This could contribute to the pronounced increase in PG levels in GPx1 kd cells but is in contrast to the equal rise of ROS levels observed herein for both kd lines (Fig. 5C), however with an assay with limited sensitivity. The amount of the PGE_2_ degradation product 15-keto-PGE_2_ was exactly the same in both kd lines (Fig. 4C), indicating that PGE_2_ turnover did not essentially contribute to differences between the kd lines. Most probably, the amount of available educts and a combination of different expression and activity levels of COX and prostaglandin synthases is responsible for the specific PG profile of the GPx1 and GPx2 kd cell lines. *Vice versa*, both isoforms could limit PG synthesis and thus reduce for example the PGE_2_/PGD_2_ ratio. Remarkably, during the resolution phase of colitis, PGD_2_ synthesis is increased which is proposed to be important for limiting inflammation [39]. In macrophages, selenium treatment enhanced GPx1 levels and in parallel increased the expression of H-PGD_2_ synthase while thromboxane synthase and mPGES-1 were downregulated by selenium [40]. These results are consistent with what has been observed here. Interestingly, GPx2 has been shown to be upregulated by the PGD_2_ metabolite, 15-deoxy-Δ^12,14^PGJ_2_ [15], which could provide an explanation why GPx2 and not GPx1 is upregulated during the regeneration of the intestinal epithelium after DSS-induced injury [15]. Altogether, GPx2 and GPx1 are important to keep the fragile prostanoid network in appropriate balance. Loss of either GPx2 or GPx1 results in exceeding inflammatory response in particular under inflammatory (IL-1β stimulated) conditions.

Besides COX-derived LMs also LOX-derived mediators were analyzed (Fig. 4) but total amounts were much lower in HT-29 cells as previously described [41]. Both 5-LOX- and 15-LOX-derived products were not considerably upregulated by IL-1β treatment (Fig. 4). In different immune cell-derived cancer cell lines, it has been shown that GPx4 [42,43] and GPx1 [44] act as important inhibitors of cellular 5-LOX activity. To generate the active Fe^3+^ form of 5-LOX, lipid hydroperoxides such as 5-HPETE are necessary, which are reduced in the presence of GPxs such as GPx1 [45]. Herein, we could show that the two putative 5-LOX products 5-HETE and 5-HEPE (Fig. 4G) were both unaffected by GPx2 kd while 5-HETE was increased in GPx1 kd cells. Although we cannot exclude that these low abundant monohydroxylated fatty acids derive from non-enzymatic autooxidation of polyunsaturated fatty acids, our finding rather confirms the described 5-LOX inhibitory effect of GPx1 and indicates a limited relevance for GPx2 in regulating 5-LOX activity. Besides this, again the available amount of educts, in this case EPA, needs to be considered. Upon loss of GPx4 either in mice or cells, 12/15-LOX-derived lipid peroxidation was strongly increased resulting in a specific form of cell death called ferroptosis [46,47]. Thus, GPx4 appears to be the main isoform that regulates this LOX. In contrast to GPx1 and 4, GPx2 is not expressed in inflammatory cells. Therefore, the inhibitory potential on enzymes of the LM network is obviously limited to epithelial cells, but could be very important to mediate cross-talk between those cell types under inflammatory conditions. Such a model has been described for GPx4 before, which is partially inhibited in intestinal epithelial cells upon *S. typhimurium* infection to allow activation of the 12-LOX pathway and to induced the recruitment of neutrophils via specific LMs [48].

By directly comparing total GPx activity of GPx1 and GPx2 kd cells towards different substrates we could show a more pronounced loss of activity in GPx1 kd cells (Fig. 5A). However, it needs to be considered that the kd was less efficient for GPx2 than GPx1 resulting in 25% of residual GPx2 but only 8% of GPx1 under + Se conditions (Fig. 3A and B). Previously, it has been discussed that GPx2 might have a higher prevalence for organic hydroperoxides [49], which however cannot be clearly answered herein. Another major difference between the two kd cell lines was the substantial downregulation of NOX1 in GPx2 kd cells, which was not observed upon loss of GPx1 (Fig. 5D). Interestingly, NOX1 has been linked to 12-LOX, whose inhibition decreased NOX1-dependent superoxide production [50]. To identify the source of H_2_O_2_ responsible for the spontaneous development of ileocolitis in GPx1/GPx2 double KO mice, these mice were crossbred with KO mice for either NOX1 or DUOX2. Almost all characteristics of double KO mice were reversible by loss of NOX1 [51], while loss of DUOX2 rescued only the inflammatory phenotype but not the higher number of apoptotic cells [52]. In contrast to GPx2 KO mice, which showed higher GPx1 expression, GPx2 kd cells obviously limit NOX1 expression, as another compensatory mechanism to reduce H_2_O_2_ production. Loss of NOX1 expression has been shown to modulate the intestinal microbiota composition and to enhance overall bacterial growth [53]. Thus, H_2_O_2_ generated by NADPH oxidases provides antimicrobial defense and, *vice versa*, loss-of-function mutations in NOX1 result in mucus layer disruption with bacterial penetration into crypts and subsequent intestinal inflammation [54]. Besides this, we have previously shown that another important mediator of mucus composition, the calcium-activated chloride channel regulator 1 (CLCA1), was decreased in GPx2 KO mice irrespective of the selenium status and associated shifts in GPx1 expression [55]. Based on that, there might be a specific function of the isoform GPx2 in modulating the immune response towards the intestinal microbiota.

## 5 Conclusion

In conclusion, we could show that both, GPx2 and GPx1, are important regulators of the LM profile of epithelial cells with likely impact on the inflammatory response. This could be ascribed to the activation of the NF-κB pathway and downstream regulation of COX-2, thereby shifting the LM profile towards pro-inflammatory PGE_2_ production by both GPx1 and 2 kd compared to the scr control. Based on these data, protective functions of GPx1 and 2 during colitis as well as inflammation-driven carcinogenesis can be anticipated.

## Funding

This work was supported by the German Research Foundation (DFG) [FOR 2558]. S. Koeberle was supported by Prochance 2018 Program Line A1 of the University of Jena [2.11.3-A1/2018-02]. A. Koeberle was supported by a Strategy and Innovation Grant from the Free State of Thuringia [41-5507-2016] and the Leibniz ScienceCampus InfectoOptics [SAS-2015-HKI-LWC]. O. Werz received funding by the DFG [SFB1127 and SFB1278].

## Declaration of competing interest

None.
